# Immunotoxins Targeting B cell Malignancy—Progress and Problems With Immunogenicity

**DOI:** 10.3390/biomedicines7010001

**Published:** 2018-12-21

**Authors:** Daniel A. Vallera, Robert J. Kreitman

**Affiliations:** 1 Laboratory of Molecular Cancer Therapeutics, Masonic Cancer Center, Department of Therapeutic Radiology-Radiation Oncology, University of Minnesota, Minneapolis, MN 55455, USA; 2 National Cancer Institute, National Institutes of Health, Bethesda, MD 20892, USA; kreitman@mail.nih.gov

**Keywords:** diphtheria toxin, pseudomonas exotoxin, immunogenicity, B-cell malignancies, CD19, CD22, deimmunized, immunotoxin, chemo-immunosuppression

## Abstract

Few immunotoxins or targeted toxins have become mainline cancer therapies. Still immunotoxins continue to be of major interest and subject of research and development as alternative therapies for drug resistant cancer. A major matter of concern continues to be immunogenicity exemplified by the anti-toxin response of the treated patient. Since some of our most effective toxins are bacterial in nature and bacterial proteins are highly immunogenic, this review describes some efforts to address this pressing issue.

## 1. Introduction

Over the years, numerous toxins have been utilized as targeted toxins mainly linked to antibody or antibody fragments. Two very important toxins are Diphtheria toxin (DT) and Pseudomonas exotoxin (PE) A. Notably, DT serves as the catalytic portion of the drug Denileukin diftitox (Ontak), the first targeted toxin approved by the FDA for the treatment of cutaneous T-cell lymphoma in 1999. Ontak was genetically engineered as a single chimeric molecule, consisting of human IL-2 spliced to truncated DT [1]. It was clinically discontinued due to production and purification issues in 2014. PE serves as the catalytic portion of the drug Moxetumomab Pasudotox (Moxe), that was approved in September 2018 by the FDA for the treatment of hairy cell leukemia (HCL). Moxe is also a chimeric molecule consisting of a single chain Fv (scFV) antibody fragment recognizing the B-cell marker CD22 spliced to truncated pseudomonas exotoxin as a single chain. These two drugs are among the very few FDA targeted toxins approved for therapy. Importantly, both DT and PE are related drugs with identical, highly specific mechanisms of action [2]. Once internalized into the target cell, both of these protein toxins catalyze ADP-ribosylation of a single amino acid on elongation factor 2 (a key protein involved in protein translation). This halts protein synthesis and triggers a series of cellular events that culminates in apoptosis of the intoxicated cell. Because these toxins act catalytically they are highly potent so one molecule gaining entry into the cytosol is sufficient to kill the cell [3]. In the case of PE, it has been calculated that fewer than 1000 molecules of immunotoxin/cell is sufficient to cause complete tumor regressions [4].

## 2. Pseudomonas Exotoxin-Based Moxetumomab

Recently, FDA approval of Moxe has validated the usefulness of this drug as an alternative therapy for drug resistant B-cell cancers. For treatment of refractory Hairy Cell Leukemia (HCL), Moxe targeting CD22 achieved 75% objective response rates in a pivotal, multicenter, open-label trial of 80 patients [5]. The most common severe side effects in <5% of patients were hypertension, febrile neutropenia, and hemolytic uremic syndrome. When compared to different PE-based targeted toxins undergoing testing for solid tumors, the highly effective nature of Moxe is believed to be due, in part, to the reduced immune status of the patients that have previously undergone treatment with Cladribine, rituxan, or many cycles of immunosuppressive chemotherapy [6]. Such treatments exert profound suppressive effects on the humoral immune system of treated patients which may prevent the formation of patient anti-toxin antibodies. This may provide evidence, albeit indirect, that deimmunization of the toxin might be highly beneficial to preventing patient antibody response. 

## 3. Diphtheria Toxin-Based DT2219

**Figure 1:**
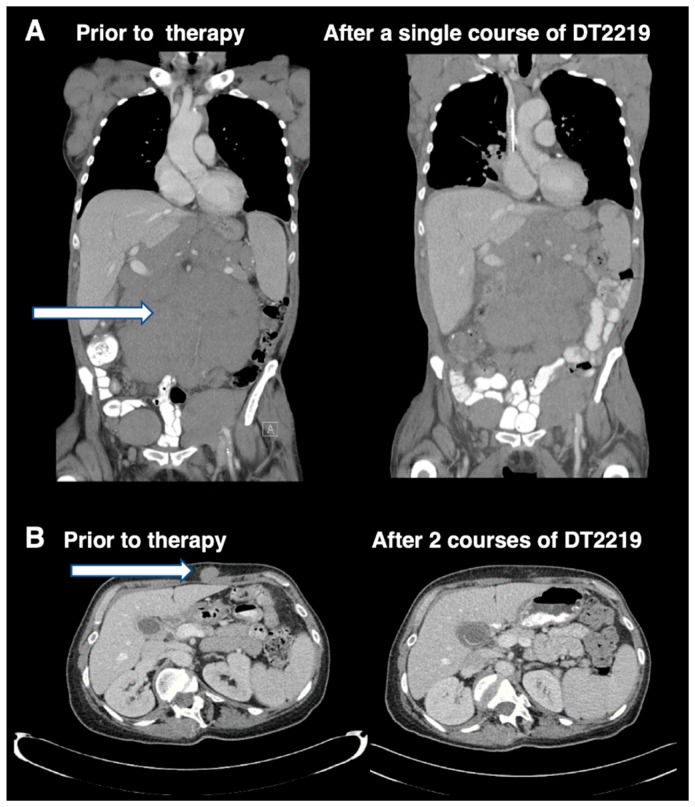
Imaging studies in patients attaining objective phase 1 study responses shown with permission of the AACR and reprinted from reference 10. Figure 1A shows an abdominal CT imaging of a 77 year-old patient with rituximab and chemotherapy-refractory CLL treated with a single course of DT2219 at dose level 40 mg/kg every other day, 4 doses before and at day 28 after therapy. A 40% reduction in the abdominal tumor mass was observed. Figure 1B shows CT images of a 53-year-old female with CD22+CD19+ relapsed, chemotherapy-refractory, marginal zone lymphoma. The patient was treated at dose level 60 mg/kg/day QOD. The patient received a second treatment course 8 weeks later, which resulted in complete resolution of the tumor mass. CT images were taken before therapy and after the second course of DT2219. Tumor mass is indicated by arrows. For greater detail, see reference 10. The image was taken at the University of Minnesota Medical Center and published with informed consent.

Despite the encouraging results obtained in CD19^+^ B-ALL patients treated with the T-cell engaging bispecific antibody blinatumomab that targets CD19, a significant percentage of patients relapsed with CD19 B-ALL after treatment [7]. Also, CAR T-cell treatment resulted in CAR-T CD19 antigen negative variants emerging at relapse. Thus, approaches that target CD19 on B-cell cancers, can result in the emergence of antigen loss variants due to immune selection pressure that brings about genetic alterations and alternatively spliced, truncated CD19 variants that account for resistance [8]. The bispecific immunotoxin DT2219 targeting both CD19 and CD22 was created to address this issue [9]. DT2219 is genetically engineered and consists of a similar truncated DT to that used in Ontak, fused genetically to an anti-CD22 scFV antibody fragment, and an anti-CD19 scFV all within the same single chain molecule. The purpose of this design was to extend the range of reactivity across a broader B cell ontological range (pro-B cell to mature B cell). Importantly, if CD19- variants arise when targeting CD19, the drug should target an entirely different B-cell marker, CD22, and thus prevent CD19^−^ variants from escaping treatment. DT2219 was tested in phase I/II studies with some responses, but published studies indicated that immunogenicity was an issue [10]. Responses are shown in Figure 1 taken from reference 10 with permission from the American Association of Cancer Research (AACR) Journals. Capillary Leak Syndrome was common among treated patients, but clinically managed. 

## 4. Deimmunization of Pseudomonas Exotoxin

Bacterial toxins are extremely immunogenic and deimmunization of these potent cytotoxic molecules has been an important goal of the targeted toxin field in order to extend their therapeutic effectiveness in humans. The Laboratory for Molecular Biology (LMB) group at National Institutes of Health (NIH) has devoted considerable effort to deimmunizing PE. To identify murine B-cell epitopes, mice were immunized to obtain monoclonal antibodies that reacted with epitopes on PE, that were then used to determine the number of epitopes [11,12]. PE was found to contain only seven major conformational epitopes localized at specific positions. The antibody defined epitopes were not spread out over the entire molecule but clustered in restricted locations over the molecules surface. The clustering enabled them to determine precise epitope locations using mutational studies of mostly amino acids with large side chains. Converting these to alanine (alanine walking) negated antibody binding. The investigators used this approach to construct a mutated recombinant immunotoxin that possessed greatly reduced B-cell immunogenicity. However, it was recognized that there are differences in immunogenicity between humans and mice, therefore a B-cell deimmunization strategy might be incomplete. 

It is now thought that the removal of T-cell epitopes is more effective at reducing immunogenicity than relying on the removal of just B-cell epitopes. Thus, the LMB used peripheral blood mononuclear cells from naive donors and cultured these cells with intact toxin to allow processing by T-cells [13]. T-cell epitopes were then identified by re-stimulating the conditioned PBMC with 15-mer peptide fragments spanning the toxin sequence. These studies indicated that PE had eight immunogenic epitopes and that other parts of the protein were non-immunogenic. Importantly, a combination of deletion of two of the amino acid epitopes and mutation by alanine conversion diminished T-cell responses significantly. Following analysis of the data, it was found that two immunogenic T-cell and two immunogenic B-cell epitopes were shared indicating that further studies on the deletion of shared epitopes are warranted. 

## 5. Deimmunization of Diphtheria Toxin

Our group devised a different approach to the deimmunization of DT. Based on the knowledge that large chain, highly hydrophilic amino acids, were mostly responsible for the immunogenicity of PE, we focused on amino acids (mostly arginine, lysine, aspartic acid, glutamine, and glutamic acid) on the molecular surface of DT pinpointed by an examination of its X-ray crystallographic structure [14]. We focused on amino acids located in prominent positions distant from the catalytic site. Several mutant combinations were bio-engineered to determine whether activity loss would be a problem. Finally, a new version of the truncated DT390 toxin was created and spliced into a DT-based targeted toxin containing seven point-mutations at crucial points with minimal catalytic activity loss. Activity loss is illustrated in Table 1 for two different immunotoxins made with deimmunized DT (dDT) and published in two different reports [14,15]. As part of these studies, immunocompetent mice were immunized with either the mutated or parental form of the toxin. Serum analysis showed a 90% reduction in anti-toxin neutralizing antibodies, even after several months, in mice immunized with the mutant, but not the parental drug form despite multiple immunizations. The drug maintained its ability to inhibit tumor growth in vivo. The experiment was repeated in a second strain of mice with a different MHC-haplotype to address whether point mutation removed T- or B-cell epitopes. Findings were identical suggesting that it was B-cell epitopes that were eliminated from DT. To further improve the deimmunization of the molecule, it will be necessary to perform T epitope mapping studies.

### 5.1. Chemo-Immunosuppression of the Immune System

Although durable remission rates have improved for many cancers, relapse is still a major problem, making multiple repeat treatment regimens necessary. Clinical testing of DT2219 revealed an unexpected low frequency of patients that developed anti-DT antibodies despite multiple injections of the drug, likely due to reduced immune status related to prior treatment with anti-B cell agents such as Rituxan or other agents [10]. This suggests that certain drug regimens might be selected that simultaneously enhance efficacy and reduce the anti-toxin response. In clinical studies of Moxe for HCL, more immediate onset of anti-toxin antibody correlated with clinical outcome. In our studies, responses were only obtained in patients that did not make neutralizing antibodies. The LMB have taken advantage of drug-induced immunosuppression in their study of PE and examined various combinations of immunotoxin and immunosuppressive therapies [6]. They evaluated an anti-mesothelin PE immunotoxin in combination with Pentostatin and Cyclophosphamide in patient studies [16]. Neutralizing antibody formation was delayed but the toxicity observed was moderately severe from known side effects of Pentostatin and Cyclophosphamide. With a different immunotoxin it was found that a Fludarabine and Cyclophosphamide combination prevented neutralizing antibody formation, blocking antibody formation after the first, second or third cycle [17]. Similar to the Pentostatin and Cyclophosphamide trial, the dose-limiting toxicities were due to side effects of the chemotherapy rather than the immunotoxin.

### 5.2. Going Forward

The discovery of localized immunogenic epitopes and our ability to reduce toxin immunogenicity by mutating them is an important step forward towards immunotoxins more effective clinical utility. Also important are the facts that key immunogenic epitopes are recognized by B-cells, key immunogenic epitopes are recognized by T-cells, and in some cases, the same epitopes are recognized by both. Together, these facts mandate further studies on the mutation of T- and B-cell epitopes. Going forward, our mutational studies of both DT and PE show that it is important to recognize that introducing mutations that reduce immunogenicity cannot be at the expense of reducing cytotoxic potency. The primary function of these enzymatic toxins is to inhibit protein synthesis and compromising cytotoxicity will reduce their anti-cancer effects. Many drugs have complementary mechanisms of action and have potent anti-cancer effects while simultaneously effecting the immune system. These drugs vary widely in mechanism including kinase inhibitors, immune checkpoint inhibitors, DNA cross-linkers, inducers of DNA intercalation, etc. The observation that these drugs or drug combinations have potential to reduce immunogenicity indicate that further combinatorial studies are warranted, but must have in vivo components that measure both efficacy and immunogenicity to be informative. We have found that mouse models are convenient indicators of efficacy but results obtained must be interpreted cautiously because of species differences between mouse and man. 

Finally, there are several reasons that immunotoxins, made with catalytic toxins, cannot be overlooked as an important class of anti-cancer drug. As previously mentioned, their catalytic mode of action, renders them extremely potent killing agents as they are capable of catalyzing reaction after reaction in the inhibition of protein synthesis at the level of the ribosome. There are published reports, attesting to the utility of immunotoxins as anti-cancer drugs capable of inducing complete regressions in mouse xenografts models (reviewed in [9]). Also, there is evidence that immunotoxins tested clinically in phase I/II clinical trials showed significant anti-tumor activity in patients [10,18,19]. A few immunotoxins, such as Moxe have been validated by FDA approval achieving impressive levels of durable clinical response against some drug refractory cancers. Recombinant immunotoxins are easily mutated for a variety of purposes that include enhancement of potency and reduced immunogenicity. Not all solutions lie in further studies of the drugs themselves and only very few studies have addressed the issue of drug delivery making this a potentially fertile area for future investigation. Just like other molecules made with small antibody fragments, immunotoxin clearance is often too rapid and more creative methods of delivery could potentially have a major impact on maintaining effective drug serum levels and subsequent therapeutic activity. We believe with the current interest in combinatorial anti-cancer approaches, mutated targeted toxins will continue to remain a valid immunotherapy focus for cancer. 

## 6. Conclusions

The approval of Moxe is an important step forward for the future of targeted toxins made with bacterial proteins. Will other drugs follow suit as alternative therapy for other types of drug refractory cancer besides B-cell malignancies? If this is to be the case, translational studies of immunogenicity must continue. More creative ways of managing the immune system in the face of potentially immunogenic drugs will be needed. 

## Figures and Tables

**Table 1:** Comparison of deimmunized versus parental diphtheria based immunotoxins.

Drug	IC50 (nM)	Induces NA	Reference
mutated deimmunized DTEGF13 (dDTEGF13)	0.03	−	[12]
parental DTEGF13	0.01	+	
Cell line control	>100	NA	
mutated deimmunized DT2219 (dDT2219)	1.0	−	[13]
parental DT2219	0.3	+	
Control anti-CD3 DT	>100	NA	

Table 1. DT was deimmunized by replacing 7 key hydrophilic amino acids in DT390 (dDT). Parental DT had no mutations. The DT DNA fragments were used to manufacture dDTEGF13 and dDT2219. dDTEGF13 consisted of dDT, human EGF and human IL-13. D2219 consisted of dDT, CD22 scFV and CD19scFV. The IC50 was determined using thymidine incorporation assays designed to measure the effects on cell proliferation. The ability of dDTEGF13 to inhibit proliferation was measured on EGF+IL13+ HT-29 colon cancer cells. The ability of dDT2219 to inhibit proliferation was measured on CD22+CD19+ Daudi cells. To measure neutralizing antibody (NA) as an indication of immune response to toxin, serum was taken from immunized immunocompetent normal mice at several time points after several immunizations. In the case of dDTEGF13 treated mice, serum was tested on HT-29 cells treated with a known inhibitory concentration of DTEGF and we tested the ability of the serum to block proliferation. In the case of serum from dDT2219 immunized mice, Daudi cells were used. Anti-CD3DT was used as an off-target control for proliferation assays since CD3 is not expressed on any of the cell lines.
